# A Multicenter, Randomized, Evaluator-Blind, No-Treatment Controlled Study of YVOIRE Y-Solution 720: A Volumizing Hyaluronic Acid Filler for Midface Volume Deficit

**DOI:** 10.1007/s00266-025-05246-4

**Published:** 2025-09-23

**Authors:** Nanze Yu, Wenyun Ting, Jinhua Xu, Wenyu Wu, Hang Wang, Jufang Zhang, Jiaming Sun, Zhiqi Hu, Sufan Wu, Xiaojun Wang

**Affiliations:** 1 Department of Plastic and Aesthetic Surgery, Peking Union Medical College Hospital, No. 41 Damucang Hutong, Xicheng District, Beijing, 100032 China; 2 Department of Dermatology, Huashan Hospital, Fudan University, Shanghai, China; 3 State Key Laboratory of Oral Diseases, Department of Cosmetic and Plastic Surgery, Oral and Maxillofacial Surgery, National Clinical Research Center for Oral Diseases, West China Hospital of Stomatology, Sichuan University, Chengdu, China; 4 Department of Cosmetic and Plastic Surgery, Hangzhou First People’s Hospital, Hangzhou, China; 5 Department of Plastic Surgery, Union Hospital, Tongji Medical College, Huazhong University of Science and Technology, Wuhan, China; 6 Department of Plastic and Aesthetic Surgery, Nanfang Hospital, Guangzhou, China; 7 Department of Plastic and Reconstructive Surgery, Center for Plastic and Reconstructive Surgery, Zhejiang Provincial People’s Hospital (Affiliated People’s Hospital, Hangzhou Medical College), Hangzhou, Zhejiang China

**Keywords:** Hyaluronic acid filler, Cross-linked HA, Soft tissue filling, Midface volume augmentation

## Abstract

**Background:**

Midface volume deficiency is a common aesthetic concern that can be addressed with dermal fillers. YVOIRE Y-Solution 720 (YYS720) is a hyaluronic acid (HA)-based filler designed for midface augmentation. This study aimed to evaluate the effectiveness and safety of YYS720 in improving midface volume.

**Methods:**

Asian participants with mild to severe midface volume loss were randomly assigned to receive either YYS720 injection (n = 173) or no treatment (control, n = 65). Midface volume was evaluated at 4, 14, and 26 weeks using Midface Volume Deficit Severity Rating Scale for Asian Faces (MFVDA-SRS) for both YYS720 and control groups. Additionally, for YYS720, midface volume was evaluated 52 weeks after the last injection.

**Results:**

At Week 26, the proportion of participants with ≥ 1-grade reduction on the MFVDA-SRS score was 82.0% (137/167 participants) for YYS720 and 5.1% (3/59 participants) for control with difference (95% confidence interval, CI) between the groups of 77.0% (66.8, 84.0), demonstrating the statistically significant improvement of YYS7720 in midface augmentation. At Week 52, the proportion of participants with ≥1-grade reduction on the MFVDA-SRS score was 76.2% for YYS720 group. Global Aesthetic Improvement Scale (GAIS) responder rates by participants were consistently high in YYS720 group across all time points up to Week 26. No specific safety concern was detected. Common treatment site responses included swelling, tenderness, and pain, which were generally mild and transient.

**Conclusions:**

This study supports that YVOIRE Y-Solution 720 is an effective and safe option for midface volume augmentation.

**Level of Evidence I:**

This journal requires that authors assign a level of evidence to each article. For a full description of these Evidence-Based Medicine ratings, please refer to the Table of Contents or the online Instructions to Authors www.springer.com/00266.

**Supplementary Information:**

The online version contains supplementary material available at 10.1007/s00266-025-05246-4.

## Introduction

The formation of wrinkles and folds in the skin is part of the natural process of aging [1, 2]. One of the main reasons for wrinkle formation is the reduction in endogenous hyaluronic acid (HA) in the skin. HA attracts and holds water; thus, with decreased HA, the skin is less hydrated and therefore less elastic, causing wrinkles and folds to form [2]. In the midface, due to decrease in HA, midface volume deficiency occurs, marked by flatter cheekbones and a sunken appearance of the paranasal area.

Dermal fillers are among the most common and effective treatments for wrinkles and folds. Recently, market demand has shifted from simple wrinkle correction to more extensive uses, such as restoring facial volume to meet aesthetic needs [3–5]. Given the effective rejuvenation provided by fillers, along with reduced downtime, lower costs, and fewer complications compared to surgical procedures, the global preference for fillers is increasing [6]. There has been a notable increase in the use of safe injectable HA fillers to correct deeper wrinkles, lift hollow cheeks, and achieve aesthetic soft-tissue augmentation in the midface [3–5, 7–10].

HA is a naturally occurring glycosaminoglycan, a very long polysaccharide composed of repeated units of disaccharides, including glucuronic acid and N-acetylglucosamine [11]. When fully hydrated, HA’s volume increases significantly, allowing it to occupy a large space. This characteristic gives HA its viscoelastic properties in solution, making it an ideal component for dermal fillers [11].

YVOIRE Y-Solution 720 (YYS720) is a sterile, cross-linked HA dermal filler manufactured by LG Chem, Ltd, which contains 20 mg/mL HA and 0.3% lidocaine. The chemical modification of HA by cross-linking significantly improves the mechanical properties of the polymer and prolongs its tissue residence time. Cross-linked HA molecules are covalently bound to each other with repeating bridges, creating a polymer network that transforms a viscous HA liquid into a gel. YYS720 uses 1,4-butanediol diglycidyl ether (BDDE), a cross-linking agent used in the majority of market-leading HA-based medical devices. The stability and biodegradability of BDDE, which has been used in these products for many years, have been well-documented [12]. However, excess amounts of cross-linking agents such as BDDE may be associated with immune-mediated adverse effects [13, 14].

Stable High-Concentration Equalized (S-HICE) cross-linking technology is used for manufacturing of YYS720, resulting in smaller, uniformly sized HA particles. These particles exhibit enhanced inter-particle interactions, leading to superior cohesivity. Simultaneously, the precisely controlled cross-linking process ensures the desired elasticity for immediate lifting upon injection. The net effect is a remarkable balance of cohesivity and elasticity achieved with significantly reduced BDDE usage [15]. This formation of a network among macromolecules of HA results in sufficiently high viscoelasticity, providing ideal duration when implanted into the skin; in addition YYS720 contains 0.3% lidocaine to provide pain relief during injection. With its high cohesivity and elasticity, YYS720 is expected to be effective in filling and maintaining volume in midface areas to improve deficiencies such as sunken cheeks.

YYS720 was approved in Republic of Korea in 2016. Since its approval, there have been no identified safety concerns or new information that would have a negative implication for the demonstrated effectiveness of YYS720 [16]. This study evaluated the effectiveness and safety of YYS720 when injected into the midface of Asian participants.

## Methods

### Study Design

This was a multicenter, randomized, evaluator-blind, no-treatment controlled study conducted in seven centers in China from November 2020 to November 2023. Eligible participants were randomly assigned in a 3:1 ratio to receive either YYS720 injection or no treatment (control) using a block randomization method.

For participants who received YYS720, an optional touch-up injection, limited to once, was allowed 4 weeks after the initial injection if the treating investigator determined that the two sides of the participant’s midface were not symmetrical, if the Midface Volume Deficit Severity Rating Scale for Asian Faces (MFVDA-SRS) score had not improved by ≥1 grade, or if the treating investigator considered a touch-up necessary.

Participants in YYS720 group were evaluated for effectiveness and safety of the treatment at 4, 14, 26, and 52 weeks after the last injection. Participants in control group were evaluated for effectiveness and safety without treatment at 4, 14, and 26 weeks after randomization.

### Study Population

Male or female participants aged between 18 and 65 years with mild to severe midface volume loss (i.e., a score of 2–4, rated using the 4-point MFVDA-SRS) were included.

Participants were excluded if they had active skin diseases, midface volume deficits due to congenital defects or trauma, or a history of hypertrophic cicatrix or keloids. Also excluded were those who had undergone or planned significant facial procedures, or had allergies to lidocaine, HA products, or Streptococcal protein. Additionally, participants who had temporary facial dermal fillers within 12 months, porcine-based collagen fillers within 24 months, or neuromodulator injections, mesotherapy, or resurfacing within 6 months before screening were excluded.

### Treatment

YYS720 is a sodium hyaluronate gel product provided in a pre-filled syringe containing 1.0 mL of colorless, clear, and viscous gel. Each 1.0 mL pre-filled syringe of YYS720 contains 20 mg/mL of sodium hyaluronate, 3 mg/mL of lidocaine hydrochloride, and phosphate-buffered saline. YYS720 was injected into the subcutaneous and/or supraperiosteal layer on both sides of the midface using the 27G disposable needles included in the package. The appropriate injection volume was determined by the investigators but was not allowed to exceed a maximum total of 6.0 mL per midface for initial and touch-up treatments combined. The recommended injection methods included tunneling, fanning, serial puncture, ferning, or crosshatching in an antegrade or retrograde manner [17]. Details for the entry point are displayed in Supplementary Fig. 1. No injectable anesthesia was used during the procedure, but lidocaine cream was applied before the injection.

### Outcome Assessments

The effectiveness of volume restoration was evaluated using the 4-point scale of MFVDA-SRS (categorized as absent [score 1], mild, moderate, or severe [score 4], as shown in Supplementary Table 1) and the Global Aesthetic Improvement Scale (GAIS; 5-grade scale, categorized as very much improved [grade 1], much improved, improved, no change, or worse than before [grade 5], as shown in Supplementary Table 2). A qualified independent blinded evaluator, who remained blinded to treatment assignments throughout the study and who was not present during the injection procedures, assessed midface volume augmentation using the MFVDA-SRS, a validated scale developed by Syneos Health, LLC, and used under a copyright licensing agreement with the sponsor (LG Chem, Ltd.). This assessment was conducted through a face-to-face interaction of the blinded evaluator and the participant (live assessment). The GAIS was evaluated by both the treating investigator (data not presented) and the participant.

The primary endpoint was the proportion of participants with a ≥ 1-grade reduction compared to baseline in the MFVDA-SRS score (i.e., MFVDA-SRS responder rate) at Week 26, based on assessment by a blinded evaluator after the last injection for YYS720 group, or randomization for control group.

Secondary endpoints included the MFVDA-SRS responder rate and GAIS responder rate (i.e., GAIS grade of “very much improved,” “much improved,” or “improved”) at Weeks 4 and 14 for both groups and Week 52 for YYS720 group, mean change from baseline in MFVDA-SRS scores at Weeks 4, 14, and 26 for both groups and Week 52 for YYS720 group, and mean change from baseline at Week 26 in overall midface volume based on measurement of 3-dimensional (3D) digital photographic images for YYS720 group. Additionally, an 11-point pain Visual Analogue Scale (VAS) was completed by participants who received YYS720, where “0” indicated “no pain” and “10” indicated “the worst pain imaginable” for both groups.

Treatment-emergent adverse events (TEAEs) were collected at each visit for safety evaluation. Common treatment site responses (CTRs, which are pain, tenderness, swelling, redness, bruising, itching, papules, and pigmentation) occurring at the injection site were evaluated during the 4-week period after the injection through participant diaries.

### Statistical Methods

All statistical analyses were performed using the SAS Version 9.4.

The sample size was calculated to achieve a power of > 0.8, ensuring at least 70% of YYS720 group were responders at Week 26, using a 1-sided exact binomial test and a 1-sided Fisher’s exact test, both with a 0.025 significance level and that the MFVDA-SRS responder rate of YYS720 group was statistically superior to that of control group using a 1-sided Fisher’s exact test with 0.025 significance level.

Demographics, baseline characteristics, total volume of the YYS720 injected, and safety endpoints were analyzed using the Safety Set, which includes all randomized participants who received YYS720 injection for YYS720 group and all randomized participants for control group. Effectiveness endpoints were analyzed using the Full Analysis Set (FAS), which includes all randomized participants who received YYS720 injection and had baseline and at least one post-baseline MFVDA-SRS score evaluation by the blinded evaluator for YYS720 group, and all randomized participants who had baseline and at least one post-baseline MFVDA-SRS score evaluation by the blinded evaluator for control group.

The primary effectiveness endpoint was tested using a 2-sided 95% exact (Clopper-Pearson) confidence interval (CI) to determine if at least 70% of the participants in YYS720 group were responders at Week 26. In addition, to prove superiority of the MFVDA-SRS responder rate of YYS720 group compared to that of control group, the lower limit of the 2-sided 95% exact CI of difference (YYS720-control) of MFVDA-SRS responder rate should have been greater than 0. The 2-sided 95% CI of rate difference was estimated by exact method of Chan and Zhang [18]. For the missing primary effectiveness variables, the Worst Observation Carried Forward approach was used, therefore, participants with a missing MFVDA-SRS score at Week 26 were imputed as non-responders.

For this effectiveness variable of mean changes from baseline, difference between the two groups and the 2-sided 95% CI of the mean difference using two-sample t-test or Wilcoxon’s rank sum test were calculated.

## Results

### Population and Treatment

**Fig. 1:**
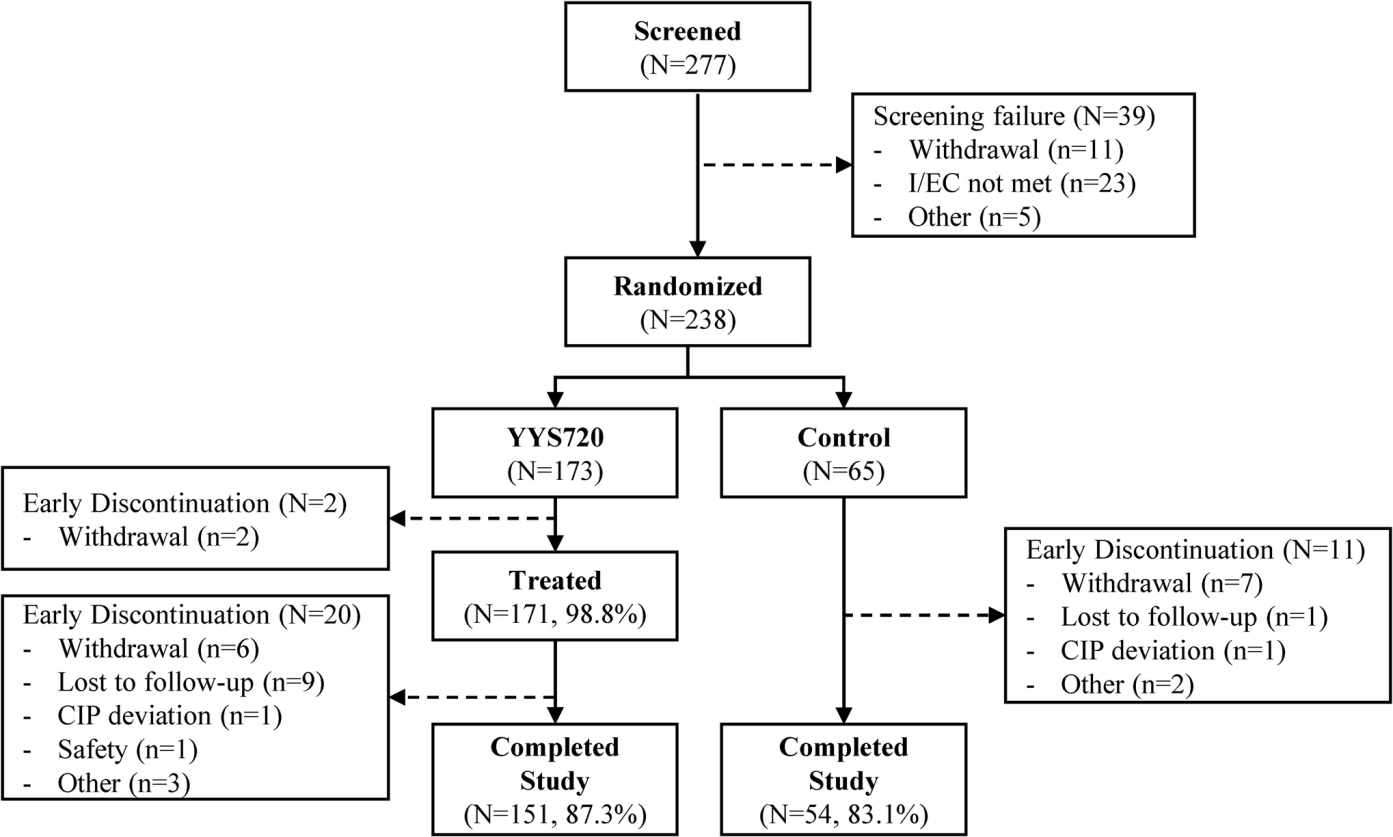
Disposition. *CIP* clinical investigation plan; *I/EC* inclusion/exclusion criteria; *YYS720* YVOIRE Y-Solution 720

A total of 277 participants were screened and 238 were randomized, 173 to YYS720 group and 65 to control group. In YYS720 group, 171 participants (98.8%) received the study treatment, and 151 participants (87.3%) completed the study up to Week 52. Of the 65 participants randomized to control group, 54 participants (83.1%) completed the study up to Week 26 (Fig. 1).

Mean age of participants in the Safety Set was 40.4 and 42.6 years in YYS720 and control groups, respectively. Female participants outnumbered male participants in both groups (88.9% in YYS720 group, 89.2% in control group). All participants were Asian. Baseline mean (±standard deviation, SD) MFVDA-SRS score was similar in the two groups (2.7 ± 0.56 for YYS720 group and 2.8±0.55 for control group) (Table 1).

**Table 1:** Demographic, baseline characteristics, and total volume injected—safety set

	YYS720 (N = 171)	Control (N = 65)
Sex		
Female, n (%)	152 (88.9)	58 (89.2)
Age (years), mean±SD	40.4 ± 10.31	42.6 ± 10.22
Asian	171 (100.0)	65 (100.0)
Baseline MFVDA-SRS score, mean±SD	2.7 ± 0.56	2.8 ± 0.55
Initial treatment volume (mL)		
n	171	–
Mean (SD)	4.2 (1.56)	–
Touch-up treatment volume (mL)		
n	58	–
Mean (SD)	1.6 (1.12)	–
Total treatment volume (mL)		
n	171	
Mean (SD)	4.7 (1.73)	
Injection technique, n (%)		
Fanning	128 (74.9)	
Tunnelling	127 (74.3)	–
Crosshatching	34 (19.9)	–
Serial puncture	23 (13.5)	–
Ferning	3 (1.8)	–

*MFVDA-SRS* midface volume deficit severity rating scale for Asian faces; *SD* standard deviation; *YYS720* YVOIRE Y-Solution 720.

In YYS720 group, all 171 participants received the initial YYS720 injection on midface, and 58 participants received the touch-up treatment. The most frequently used injection techniques were fanning (128 participants, 74.9%) and tunnelling (127 participants, 74.3%). The mean total volume of YYS720 injected for the midface on both sides was 4.7 mL (n = 171), combining a mean initial volume of 4.2 mL (n = 171) and a mean touch-up volume of 1.6 mL (n = 58) (Table 1).

### Effectiveness

#### Primary Endpoint

**Fig. 2:**
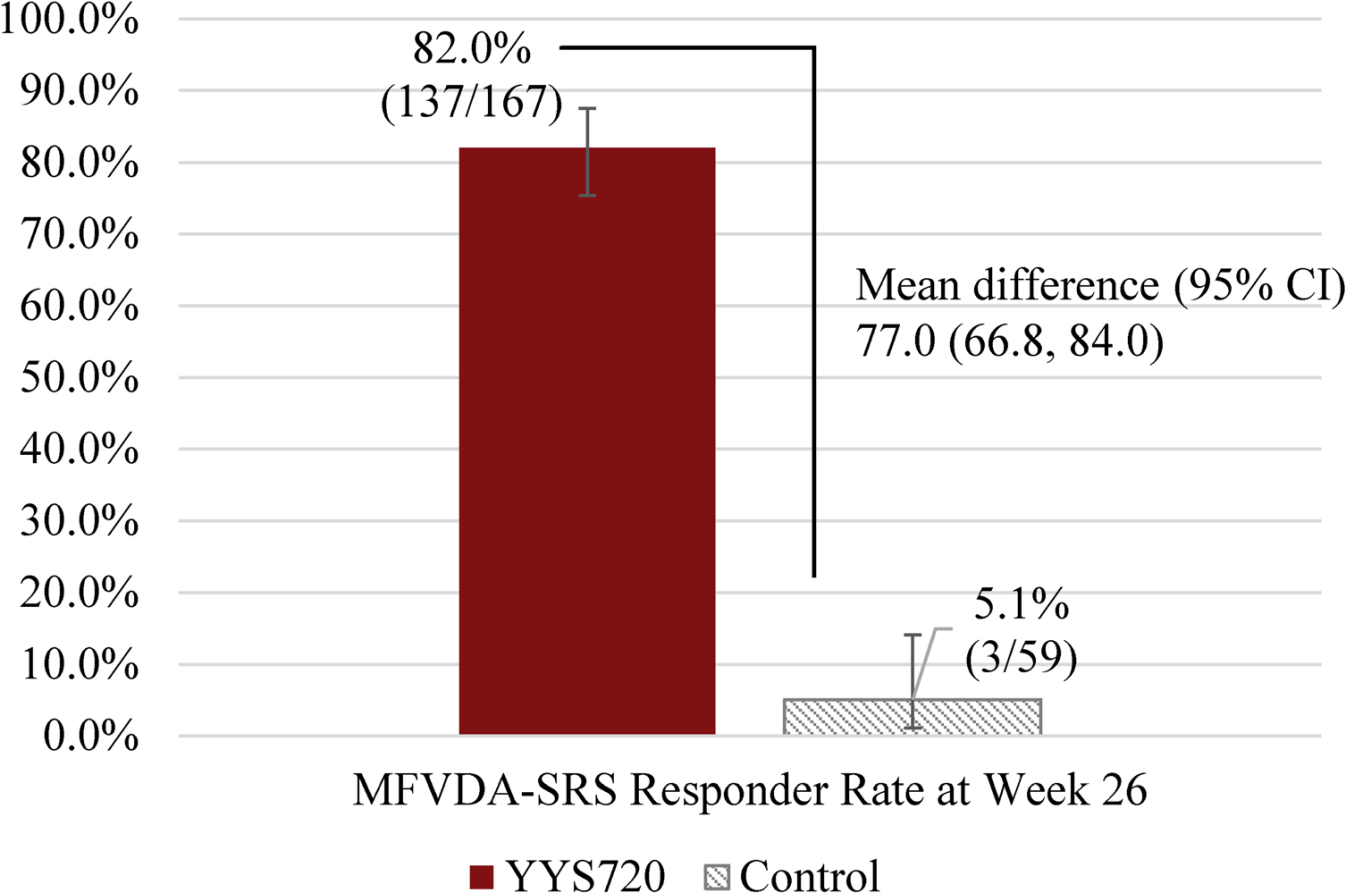
MFVDA-SRS responder rate^a^ (95% CI) at week 26 by blinded evaluators – full analysis set. CI, confidence interval; MFVDA-SRS, midface volume deficit severity rating scale for Asian faces; YYS720, YVOIRE Y-Solution 720. ^a^The response rate was calculated based on the proportion of participants with a ≥ 1-grade reduction on the MFVDA-SRS score evaluated by independent blinded evaluator at week 26, compared to that at baseline. Missing data were imputed as non-responders

**Fig. 3:**
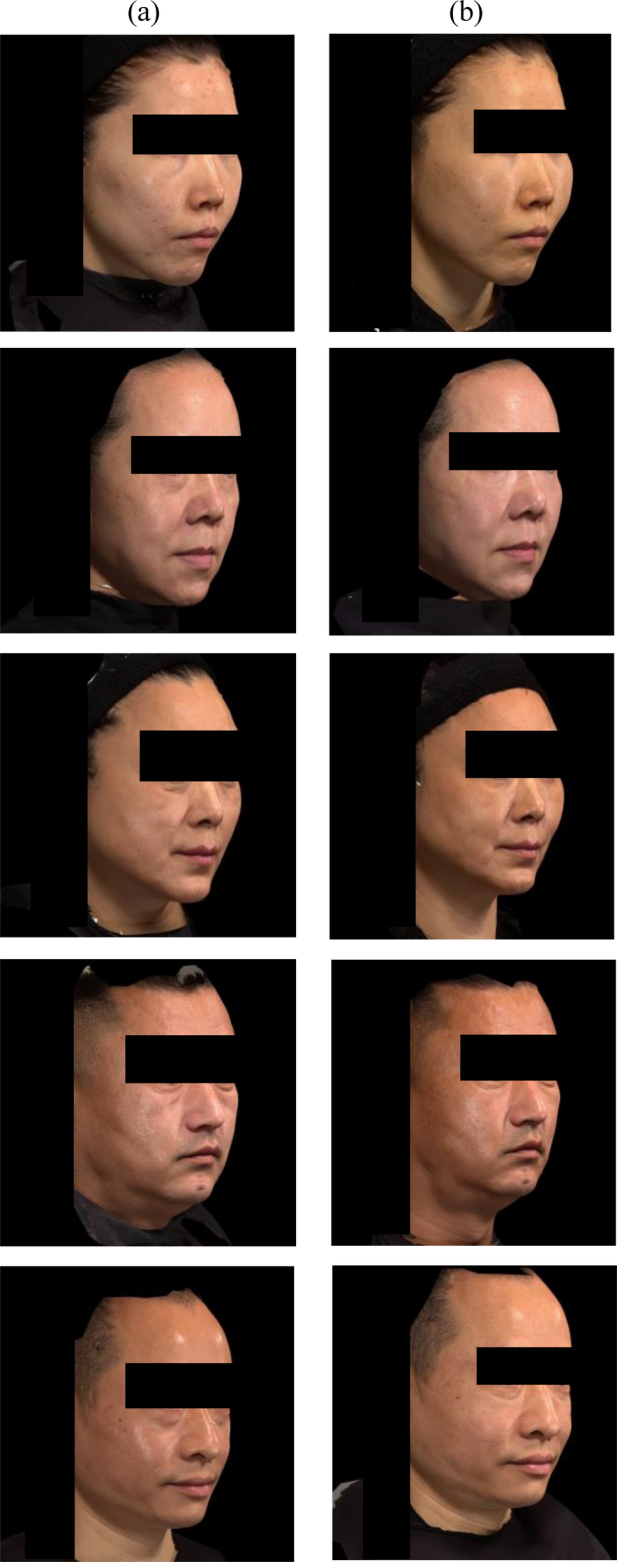
Photograph of representative participants **a** before YYS720 injection **b** 26 weeks after YYS720 injection. *YYS720* YVOIRE Y-Solution 720

After the injection of the study treatment, the MFVDA-SRS responder rates at Week 26 were 82.0% (137/167 participants) for YYS720 group and 5.1% (3/59 participants) for control group. The Clopper-Pearson 2-sided 95% exact CI’s lower limit was 75.4%, which is above 70%, demonstrating that at least 70% of the participants in YYS720 group were responders at Week 26. The difference in the MFVDA-SRS responder rate at Week 26 between the two groups was 77.0%, with a 95% CI of 66.8 to 84.0%, demonstrating the statistical superiority of YYS720 group compared to control group (Fig. 2). Figure 3 presents the representative photographs of five participants taken before and 26 weeks after YYS720 injection.

#### Secondary Endpoints

The MFVDA-SRS responder rate was 92.0% (149/162 participants) and 3.6% (2/56 participants) at Week 4 for YYS720 and control group, respectively, and 88.3% (136/154 participants) and 5.4% (3/56 participants) at Week 14 for YYS720 and control groups, respectively. The differences between the two groups (95% CI) were 88.4% (79.5, 93.6) at Week 4 and 83.0% (72.5, 89.8) at Week 14. At Week 52, the MFVDA-SRS responder rate in YYS720 group was 76.2% (115/151 participants) (Supplementary Fig. 2).

**Fig. 4:**
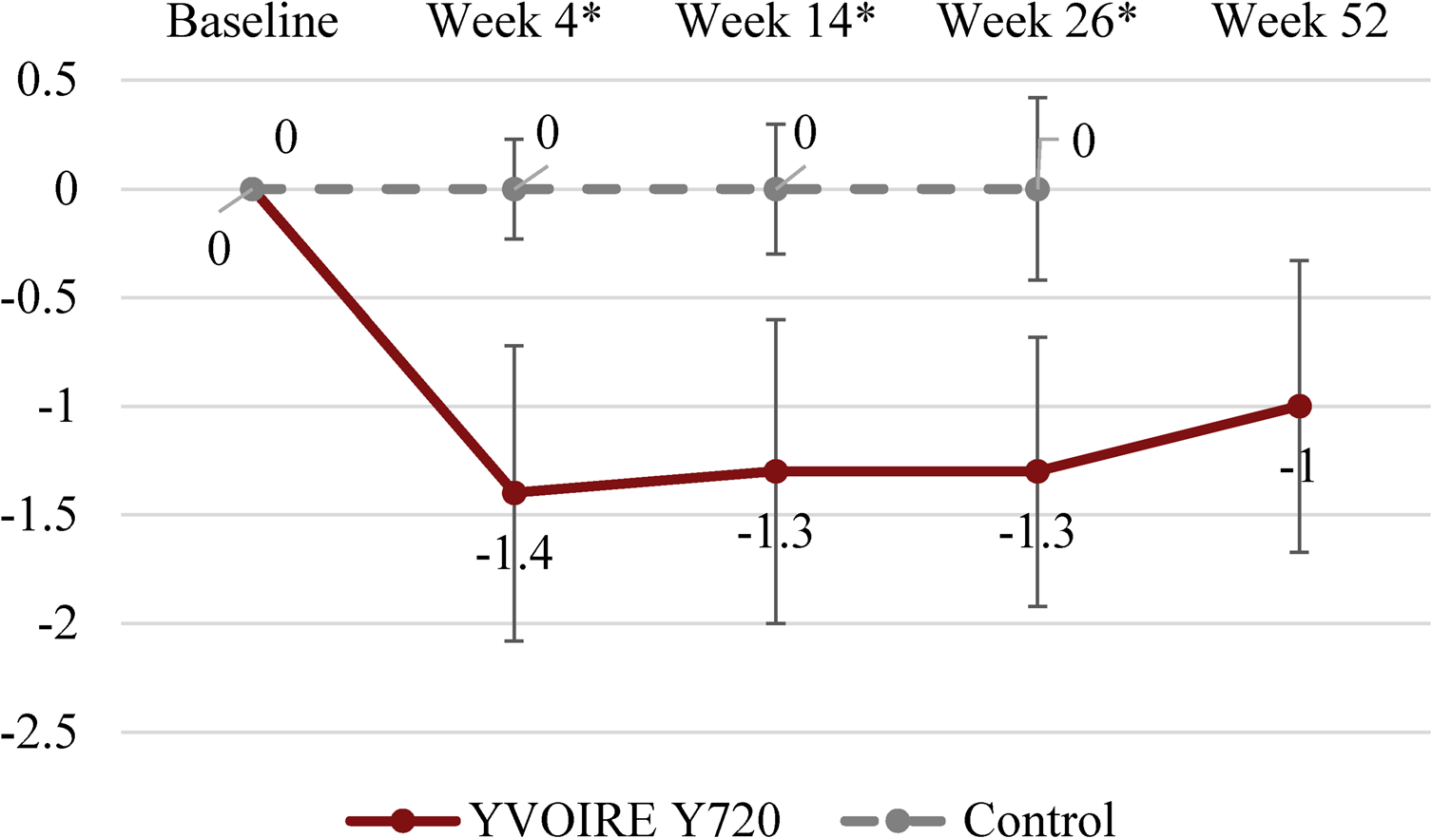
Mean (SD) MFVDA-SRS score change from baseline over time by blinded evaluators—full analysis set. SD, standard deviation; MFVDA-SRS, midface volume deficit severity rating scale for Asian faces; YYS720, YVOIRE Y-Solution 720. *Statistically significant difference between the groups (*p *< 0.001)

Decreases from baseline MFVDA-SRS score were seen at Week 4 (−1.4 ± 0.68), 14 (−1.3 ± 0.70), 26 (−1.3 ± 0.62), and 52 (−1.0 ± 0.67) in YYS720 group but not in control group at Week 4, 14 and 26. The differences in the mean changes from baseline between the two groups at Weeks 4, 14, and 26 were statistically significant (*p *< 0.001) (Fig. 4).

**Fig. 5:**
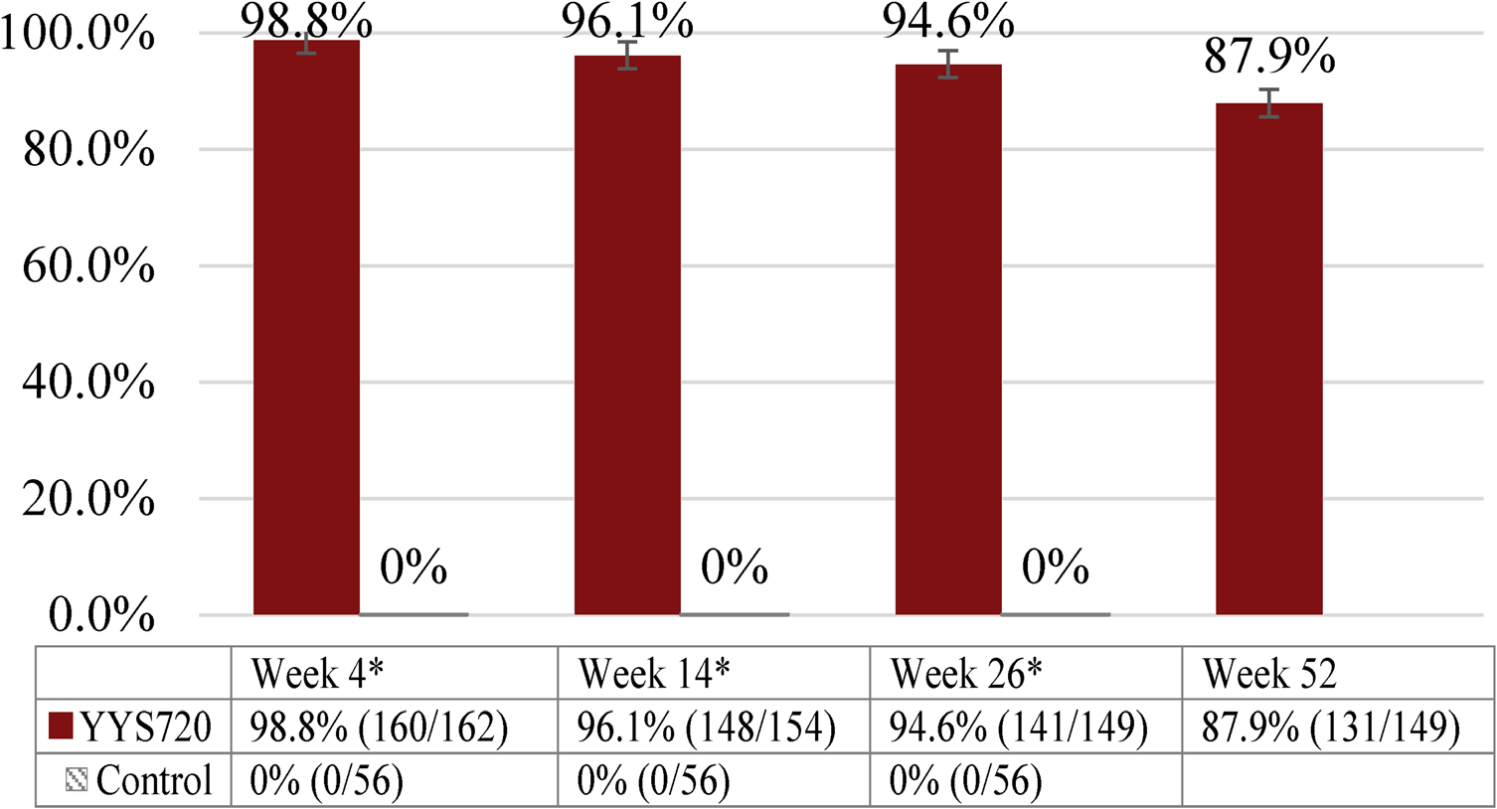
GAIS responder rate^a^ by participants—full analysis set. ^a^The response rate was calculated based on the GAIS evaluation by participants at each visit. *Statistically significant difference between the groups

The GAIS responder rates as assessed by the participants were 98.8% (160/162 participants), 96.1% (148/154 participants), 94.6% (141/149 participants), and 87.9% (131/149 participants) at Week 4, 14, 26, and 52, respectively, in YYS720 group. No participants in control group were responders in this assessment (Fig. 5).

The mean changes from baseline at Week 26 in overall midface volume based on measurement of 3D digital photographic images were 5.1 ± 3.84 and −1.1 ± 3.93 cm^3^ in YYS720 and control groups, respectively; the difference between the two groups was statistically significant (*p* < 0.001).

The mean pain VAS score evaluated by participants was 2.6 ± 1.61 (n = 167) for the initial treatment and 2.6 ± 1.69 (n = 57) for the optional touch-up treatment.

### Safety

During the 26-week study period, TEAEs were reported in 19.9% and 18.5% of participants in YYS720 and control groups, respectively. Most of TEAEs were mild in intensity. There were no statistically significant differences between the two groups with regard to the frequency of TEAEs during the 26-week study period, including study device or treatment procedure related TEAEs, serious TEAEs, and TEAEs leading to study withdrawal (*p* > 0.05) (Supplementary Table 3). During the 52-week study period, in YYS720 group TEAEs were reported in 42.7% of participants, with most frequently reported preferred terms (PTs) of COVID-19 (17.5%), upper respiratory tract infection (4.1%), and urinary tract infection (3.5%).

Three participants in YYS720 group reported treatment-related TEAE: hypoaesthesia, acne, and embolism venous, all of which were reported during the 26-week study period and mild in intensity. One patient withdrew from the study due to the treatment-related AE (PT: embolism venous). Three serious TEAEs (PTs: supraventricular tachycardia, anal fistula, and cervicobrachial syndrome) were reported in three participants in YYS720 group and none were considered related to the study device or treatment procedure. All six events noted above resolved during the study period. No deaths were reported.

Most participants who received YYS720 reported CTRs. The most frequently reported CTRs were swelling (91.0%), tenderness (86.2%), and pain (79.0%) for the initial injection. For the touch-up injection, tenderness (72.7%), swelling (70.9%), and pain (67.3%) were most frequently reported. Most CTRs were considered mild in intensity, and the mean duration of all CTRs for the initial injection ranged from 3.5 to 11.4 days and 5.2 to 9.1 days for the touch-up injection (Table 2). Most CTRs occurred shortly after treatment, and the occurrence rate decreased over 4 weeks of post-treatment.

**Table 2:** Common treatment reaction that occurred within 4 weeks after the initial and touch-up injection—safety set

	YYS720 (N=171)	
	Initial injection (N = 171)	Touch-up injection (N = 58)
Participants with CTR, n (%)^a^	161 (96.4)	46 (83.6)
Redness	116 (69.5)	27 (49.1)
Pain	132 (79.0)	37 (67.3)
Tenderness	144 (86.2)	40 (72.7)
Swelling	152 (91.0)	39 (70.9)
Bruising	116 (69.5)	28 (50.9)
Itching	39 (23.4)	11 (20.0)
Papule	23 (13.8)	3 (5.5)
Pigmentation	30 (18.0)	6 (10.9)
Duration of CTR, mean (SD)		
Redness	3.5 (3.62)	5.2 (5.18)
Pain	6.1 (5.96)	5.6 (5.24)
Tenderness	10.6 (8.45)	9.1 (7.45)
Swelling	10.1 (11.61)	7.5 (5.00)
Bruising	11.0 (11.01)	8.4 (5.03)
Itching	5.2 (4.55)	5.5 (6.76)
Papule	6.9 (8.41)	7.0 (3.00)
Pigmentation	11.4 (19.41)	6.2 (4.22)

*CTR* common treatment reaction; *SD* standard deviation; *YYS720* YVOIRE Y-Solution 720

^a^The percentage was based on participants who received the initial (n = 167) or touch-up injection (n = 55) and had their CTR diaries collected post-treatment

## Discussion

This study evaluated the effectiveness and safety of YYS720, a cross-linked HA dermal filler, when injected into the midface of Asian participants. YYS720 group achieved a significantly higher MFVDA-SRS responder rate at Week 26, as evaluated by an independent blinded evaluator, compared to control group, demonstrating the effectiveness of YYS720 in improving midface volume deficiency. Despite the limitation that these results are not being compared head-to-head, the responder rates were higher (82%) than the 76% improvement rate observed for midface volume at Month 6 after injection of a different HA filler product in Asian population in a study with similar design [19]. This higher responder rate may be attributed to the well-balanced cohesiveness and elasticity of YYS720, which helps maintain its intended shape and functionality over time.

The secondary endpoints further supported these findings, with significant improvements in MFVDA-SRS responder rates at Weeks 4 and 14 and GAIS responder rates assessed by the participant at Weeks 4, 14, and 26. GAIS responder rates assessed by the participant of 94.6% at Week 26 align closely with findings from other multicenter trials of HA fillers in Asian population, which reported GAIS scores ranging from 93.8 to 98.8% at comparable time points [20–22]. These consistent results across studies reinforce the reliability of HA fillers for midface augmentation and support the clinical relevance of our findings. In addition, this study provides long-term data on YYS720 up to 52 weeks, which is less commonly reported, as many studies are designed to evaluate the efficacy of HA fillers over shorter durations, such as 6 months [19–21]. The sustained MFVDA-SRS responder rate of 76.2% and GAIS responder rate of 87.9% at Week 52 highlight the durability of YYS720’s volumizing effect, which is a key consideration for both patients and clinicians.

The safety profile of YYS720 was favorable, with a TEAE incidence rate of 19.9% during the 26-week study period and no serious TEAE or deaths considered to be related to study device or treatment procedure. During the 52-week study period, in YYS720 group the incidence rate of TEAEs was 42.7%. However, the most frequently reported PTs were COVID-19 (17.5%), upper respiratory tract infection (4.1%), and urinary tract infection (3.5%) in YYS720 group. Given that these were the most frequently reported terms from 26 to 52 weeks of the study period, and considering the study was conducted during the COVID-19 pandemic, it is important to focus on TEAEs considered related to the study device or treatment procedure.

The incidence of TEAEs in YYS720 group considered to be related to the study device or treatment procedure was low (three events, 1.8%) and all three events were reported during the 26-week study period. Hypoaesthesia, acne, and venous embolism are complications that could occur after dermal HA injection due to nerve injury, inflammation, vascular damage, and possibly other factors, as risks associated with intradermal injections recognized by the European Union's Common Specifications [23]. However, all three events were mild in intensity. In the case of venous embolism that led to study withdrawal, it occurred after the touch-up injection, and hyaluronidase was administered to treat the event. All three events, including the venous embolism, were resolved during the study period.

Almost all participants who received YYS720 injection reported CTRs, however these were generally mild and transient, resolving within a few days. The high incidence of CTRs shortly after injection and their subsequent decrease over time is typical for injectable treatments and aligns with the expected safety profile of HA fillers [9, 24].

This study has several limitations. First, it was only conducted in a Chinese population, which may affect the generalizability of the findings to other ethnic groups. Second, the use of a no-treatment control group may limit the interpretability of comparative effectiveness, as improvements in the treatment group were expected. However, this design was chosen to isolate the specific effects of YYS720 from natural variation or placebo response and to quantify the magnitude and durability of midface volume restoration using YYS720. Future studies may benefit from including active comparators to further contextualize clinical utility. Additionally, due to the nature of the study design, participants and treating investigators were aware of whether the participants received the study treatment, which could affect the GAIS evaluation by participants. To mitigate this, the primary effectiveness endpoint was evaluated by an independent blinded evaluator to minimize or avoid bias.

In addition to HA fillers, other treatment options for midface volume restoration include surgical options such as malar implants and autologous fat grafting. Malar implants offer a permanent solution by augmenting the cheekbone structure, but they require invasive surgery and carry risks such as infection, implant displacement, and asymmetry. Autologous fat grafting involves procuring fat from the patient’s own body and injecting it into the midface. While it is biocompatible and can provide natural-looking results, its long-term efficacy is variable due to unpredictable fat resorption rates [25].

Non-resorbable fillers, such as polymethylmethacrylate and polyacrylamide gel, have also been used for facial volume restoration. These fillers offer long-lasting effects but are associated with higher risks of complications, including granuloma formation, migration, and difficulty in removal if adverse events occur [25].

Given these considerations, HA fillers like YYS720 provide a favorable balance between effectiveness, safety, and reversibility, making them a preferred choice for many patients and clinicians.

## Conclusion

In conclusion, YYS720 demonstrated significant effectiveness in improving midface volume deficiency with no notable safety findings in Asian participants. The high responder rates, sustained effectiveness up to 52 weeks after the last injection, and low incidence of serious AEs support the use of YYS720 as a reliable option for midface augmentation.

## Supplementary Information

Below is the link to the electronic supplementary material.

Supplementary file1 (DOCX 1127 KB)
