# Supplementary material for: A Multicenter, Randomized, Evaluator-Blind, No-Treatment Controlled Study of YVOIRE Y-Solution 720: A Volumizing Hyaluronic Acid Filler for Midface Volume Deficit
Source: Aesthetic Plast Surg. 2025 Sep 23;50(3):776–85. doi: 10.1007/s00266-025-05246-4 (PMC12992433; doi:10.1007/s00266-025-05246-4)
Supplement: Supplementary file 1 — Supplementary file1 (DOCX 1127 KB) [file 266_2025_5246_MOESM1_ESM.docx]

Supplementary Information

Supplementary Table 1. Midface Volume Deficit Severity Rating Scale (MFVDA-SRS) for Asian Faces

| **Score 1 – Absent** | **Score 2 – Mild** | **Score 3 – Moderate** | **Score 4 – Severe** |
| --- | --- | --- | --- |
| - Very full, round face with no contour or volume deficiencies in the overall mid face. | - Mild volume deficiency in the overall mid face with slight flattening in the anteromedial cheek, zygomaticomalar, and/or submalar regions. - May have presence of mild tear troughs and/or nasolabial folds. | - Moderate volume deficiency in the overall mid face, including the anteromedial cheek, zygomaticomalar, and/or submalar regions. - May have presence of moderate tear troughs and/or nasolabial folds. | - Severe volume deficiency in the overall mid face with concavity and severe hollowing in the anteromedial cheek, zygomaticomalar, and/or submalar regions. - May present with significant tear troughs and/or nasolabial folds. |

**Supplementary Table 2. Global Aesthetic Improvement Scale**

| **Score** | **Grade** | **Description** |
| --- | --- | --- |
| 1 | Very much improved | Optimal cosmetic results for the implant in this subject. |
| 2 | Much improved | Marked improvement in appearance from initial condition, but not completely optimal for this subject. |
| 3 | Improved | Obvious improvement in appearance from the clinical condition. |
| 4 | No change | The appearance is essentially the same as the original condition. |
| 5 | Worse than before | The appearance is worse than the original condition. |

**Supplementary Table 3. Adverse Events during the Study Period – Safety Set**

|  | **YYS720**  **(N=171)** | | **Control**  **(N=65)** | **P-value^b^** |  |
| --- | --- | --- | --- | --- | --- |
|  | **During 26-week** | **During 52-week** | **During 26-week** |  |  |
| Participants with TEAEs^a^, n (%) |  |  |  |  |  |
| TEAEs | 34 (19.9) | 73 (42.7) | 12 (18.5) | NS |  |
| Study device or treatment procedure related TEAEs | 3 (1.8) | 3 (1.8) | 0 | NS |  |
| TESAEs | 3 (1.8) | 3 (1.8) | 0 | NS |  |
| TEAEs leading to discontinuation | 1 (0.6) | 1 (0.6) | 0 | NS |  |
| Death | 0 | 0 | 0 | - |  |
| AE, adverse event; IMD, investigational medical device; TEAE, treatment-emergent adverse event; TESAE, treatment-emergent serious adverse event.  ^a^ A TEAE is any AE that occurs or worsens on or after the first IMD injection in treatment group. For control group, a TEAE is any AE that occurs or worsens on or after the randomization.  ^b^ Comparison of YYS720 group with control group for the 26-week study period was performed using Chi-square test or Fisher exact test. | | | | | |

**Supplementary Figure 1. Entry Point for Injection**


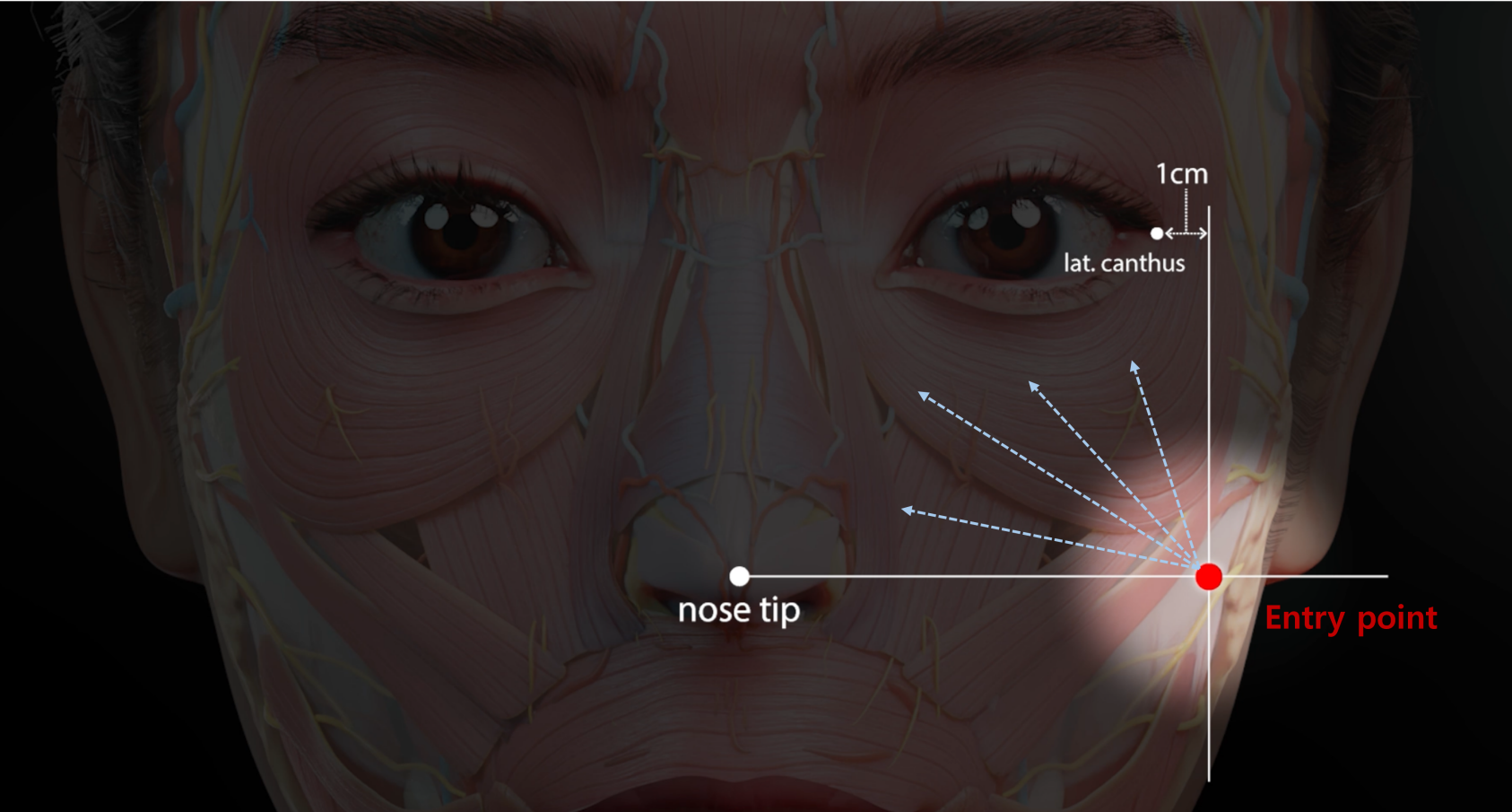


**Supplementary Figure 2. MFVDA-SRS Responder Rate^a^ (95% CI) Over Time by Blinded Evaluators – Full Analysis Set**

^a^ The response rate was calculated based on the proportion of participants with a ≥1-grade reduction on the MFVDA-SRS score evaluated by blinded evaluator results at each visit 26, compared to that at baseline. .

*Statistically significant difference between the groups.
